# Celastrol elicits antitumor effects by inhibiting the STAT3 pathway through ROS accumulation in non-small cell lung cancer

**DOI:** 10.1186/s12967-022-03741-9

**Published:** 2022-11-12

**Authors:** Zhucheng Zhao, Yanmao Wang, Yuyan Gong, Xian Wang, Luyao Zhang, Haiyang Zhao, Jifa Li, Jiandong Zhu, Xiaoying Huang, Chengguang Zhao, Lehe Yang, Liangxing Wang

**Affiliations:** 1 The First Affiliated Hospital, Wenzhou Medical University, Wenzhou, 325000 Zhejiang China; 2 School of Pharmaceutical Sciences, Wenzhou Medical University, Building 11, Chashan Street, University Town, Wenzhou, 325035 Zhejiang China; 3 Affiliated Yueqing Hospital, Wenzhou Medical University, Wenzhou, 325600 Zhejiang China; 4 The Institute of Life Sciences, Wenzhou University, Wenzhou, 325035 Zhejiang China; 5 Department of Oncology, Jianli People’s Hospital, Jingzhou, 433300 Hubei China

**Keywords:** Celastrol, STAT3, ROS, NSCLC, Inhibitor

## Abstract

**Background:**

Non-small cell lung cancer (NSCLC) is the most common lung cancer with high mortality across the world, but it is challenging to develop an effective therapy for NSCLC. Celastrol is a natural bioactive compound, which has been found to possess potential antitumor activity. However, the underlying molecular mechanisms of celastrol activity in NSCLC remain elusive.

**Methods:**

Cellular function assays were performed to study the suppressive role of celastrol in human NSCLC cells (H460, PC-9, and H520) and human bronchial epithelial cells BEAS-2B. Cell apoptosis levels were analyzed by flow cytometry, Hoechst 33342, caspase-3 activity analysis, and western blot analysis. Intracellular reactive oxygen species (ROS) were analyzed by flow cytometry and fluorescence microscope. Expression levels of endoplasmic reticulum (ER) stress-related proteins and phosphorylated signal transducer and activator of transcription 3 (P-STAT3) were identified via western blot analysis. A heterograft model in nude mice was employed to evaluate the effect of celastrol in vivo.

**Results:**

Celastrol suppressed the growth, proliferation, and metastasis of NSCLC cells. Celastrol significantly increased the level of intracellular ROS; thus, triggering the activation of the ER stress pathway and inhibition of the P-STAT3 pathway, and eventually leading to cell apoptosis, and the effects were reversed by the pre-treatment with N-Acetyl-l-cysteine (NAC). Celastrol also suppressed tumor growth in vivo.

**Conclusion:**

The outcomes revealed that celastrol plays a potent suppressive role in NSCLC in vitro and in vivo. Celastrol induces apoptosis via causing mitochondrial ROS accumulation to suppress the STAT3 pathway. Celastrol may have potential application prospects in the therapy of NSCLC.

**Supplementary Information:**

The online version contains supplementary material available at 10.1186/s12967-022-03741-9.

## Background

Lung cancer is one of the most common cancers in the world [1]. Non-small cell lung cancer (NSCLC) accounts for about 85% of all lung cancers, and it includes lung adenocarcinoma (LUAD), lung squamous cell carcinoma (LUSC), and lung large cell cancer (LCLC) [2]. According to the latest annual report of global cancer statistics, NSCLC still occupies an important position in cancer-related deaths worldwide [3]. Although the progress in surgical treatment, systemic chemoradiotherapy, targeted therapy, and immunotherapy is gradually improving the clinical treatment effect in NSCLC patients, the prognosis is still not ideal [4, 5]. The five-year survival rate of patients with early NSCLC is still very low; only 15%, and the progression of cancer is almost inevitable. Currently, good treatment measures are not available for patients with advanced NSCLC [6, 7]. Therefore, it is very important to clarify the molecular mechanism of the occurrence and development of NSCLC, search for more specific and effective therapeutic targets, and develop new therapeutic drugs.

Reactive oxygen species (ROS) mainly include hydroxyl radical (OH–), superoxide anion radical (O_2_–), and hydrogen peroxide (H_2_O_2_), which are the by-products in the process of the cellular aerobic metabolism [8]. Studies have shown that tumor cells have a higher basic level of ROS than normal cells due to their high metabolic status, mitochondrial dysfunction, and abnormal activation of oncogenes [9]. The antioxidant system in tumor cells can prevent the excessive accumulation of ROS and maintain the redox balance [10]. When the antioxidant system in cells is ineffective in removing excessive ROS, oxidative stress will increase and tumor cell apoptosis and autophagy will be induced. At this time, the toxic level of ROS will play an anti-tumor role [11]. High ROS levels in tumor cells also make them more vulnerable to cell damage caused by externally induced increases in ROS. In recent years, it has been confirmed that one of the mechanisms of the anti-tumor effects of some drugs, such as paclitaxel [12] and trienol [13], is to induce cells to produce toxic ROS. Therefore, therapy that amplifies ROS in tumor cells to toxic levels may be an effective anti-tumor approach.

Endoplasmic reticulum (ER) stress is a response process, in which cells respond to the accumulation of misfolded and unfolded proteins and calcium imbalance in the endoplasmic reticulum, and activate the unfolded protein response (UPR), ER overload response, and caspase-12 mediated apoptosis pathway [14]. ER stress occurs when ROS, hypoxia, or nutrient deficiency causes disruption of cellular redox regulation and imbalance of cellular homeostasis. In tumor cells, during the early stage of ER stress, the UPR can regulate protein folding and degrade unfolded proteins to maintain cell homeostasis [15]. When ER stress persists and the UPR cannot deal with unfolded proteins, ER stress can be mediated by the activating transcription factor 6 (ATF6), inositol requiring enzyme 1 (IRE1), and protein kinase R (PKR)-like endoplasmic reticulum kinase (PERK) signaling pathways that transduce downstream apoptotic pathways to induce apoptosis [16].

Signal transducer and activator of transcription 3 (STAT3) is an important transcription factor. It plays an important role in cell functions, such as cell growth, survival, differentiation, metabolism, host defense, and immune regulation [17]. A large amount of research evidence has shown that abnormal activation of STAT3 occurs during the development, drug resistance, and metastasis of various human tumors, including NSCLC [18]. Inhibition of STAT3 activity has become an important way to prevent tumor occurrence and metastasis [19]. Relevant studies have reported that in prostate cancer [20] and gastric cancer [21], excessive accumulation of ROS can inhibit the STAT3 signaling pathway and exert an anti-tumor effect. Therefore, inhibition of the STAT3 signaling pathway by increasing ROS levels in tumor cells may be a new target for anti-tumor drugs.

Celastrol is the main bioactive ingredient extracted from traditional Chinese medicinal herb Tripterygium wilfordii [22]. Studies have shown that celastrol has potential cytotoxicity in a variety of tumor cells [21–23]. Recent studies have also found that celastrol can significantly increase the intracellular ROS levels, leading to apoptosis, cell cycle arrest, and autophagy of cancer cells [24, 25]. However, the antitumor mechanism of celastrol in NSCLC has not been fully understood. In this study, the effects of celastrol on proliferation, migration, invasion, and apoptosis of NSCLC cells were observed. The effects of celastrol on the ROS level, ER stress level, and STAT3 signaling pathway in NSCLC cells were observed. Finally, a xenograft tumor model of NSCLC in nude mice was constructed to observe the effect of celastrol on NSCLC in vivo*;* thus, providing a new research basis for celastrol as a potential drug for the treatment of NSCLC.

## Methods

### Cell lines and cultivation

H460, PC-9, H520 and BEAS-2B lineage cells were obtained from the Cellular Resources Center of the SIBS (CAS, PRC). PC-9 cells were cultivated in Dulbecco’s Modified Eagle Medium (DMEM) (Thermo Fisher Scientific) with 10% fetal bovine serum (FBS) (TFS). H460, H520 and BEAS-2B cells were cultivated in RPMI-1640 intermediary (TFS) with 10% FBS (TFS). The environment (5% CO_2_, 37 °C) was provided for cell incubation.

### Antibodies and reagents

Celastrol was supplied by Baoji Herbest Bio-Tech Co., Ltd. Media were provided by Invitrogen (America). Antisubstances of ATF4, phosphorylated eukaryotic initiation factor 2 alpha (P-EIF2α), EIF2α, STAT3, and glyceraldehyde 3-phosphate dehydrogenase (GAPDH) were supplied by Cell Signaling Technology (USA). Antisubstances, P-STAT3 and BCL2 Associated X (BAX), were provided by Abcam Co. (USA). Antibodies of B-cell lymphoma-2 (BCL-2) and secondary antibodies were purchased from Santa Cruz Biotechnology Inc. (USA). The Overall Protein Abstraction Kit was supplied by Boster Biological Technology (PRC). Dimethylsulfoxide (DMSO) and methylthiazolyldiphenyltetrazolium bromide (MTT) were provided by Sigma-Aldrich Co. (USA). Apoptosis kits were purchased from BD Pharmingen (USA). The Caspase 3 Assay Kit was purchased from Abcam Co. (Cambridge, MA, USA). ROS probes 2′,7′-Dichlorodihydrofluorescin diacetate (DCFH-DA) and N-Acetyl-l-cysteine (NAC) were obtained from Beyotime Biotech (Nantong, China).

### MTT cell toxicity assay

Cell viability and cytotoxicity in mankind NSCLC cells were identified by MTT assay. Cells (4–5 × 10^3^ per well) were seeded into plates with 96 wells. Then, different concentrations of celastrol were added into the plates for 48 h. Further, 25 μL MTT solution was added to the plates for 4 h before the MTT assay. Formazan crystals were dispersed in 150 µL DMSO and the optical density (OD) was identified via a Micro plate Reading device at 490 nm. The half-maximal inhibitory concentration (IC_50_) data were identified via GraphPad Pro Prism 7.0.

### Colony formation analysis

The colony formation activity was assessed by the colony forming analysis. Cells were implanted into 6-well plates in 5% CO_2_ atmosphere at 37 °C overnight. Celastrol (1, 2, and 4 μM) and DMSO were supplemented into the plates for 24 h. Further, 7–14 days later, when colonies were macroscopic, the plate was cleaned with phosphate-buffered saline (PBS), treated with 4% paraformaldehyde (PFA) fixation under room temperature (RT) for 15 min, then cleaned with pure water 3 times and it was dyed with Gentian violet for 10 min.

### Wound-healing migration analysis

Wound-healing analysis was employed to assess the capability of cell motility. The cells were grown to about 80–90% confluence in plates with 6 wells, and afterwards, scraping of the cell mono-layer was performed at the center of the wells with a bacteria-free 10 μL pipet to create a scratched region. After that, the plates were cleaned in PBS to remove the scratched cells. Subsequently, the cells were cultivated in RPMI intermediary (without sera) with vector control or celastrol (1, 2, and 4 μM). Leica digital camera (Tokyo, Japan) was employed for microphotographs of cells that crossed the wound area.

### Transwell assay

Transwell analysis was employed to assess the cellular invasion capability. The cells were seeded into a transwell chamber at 2–4 × 10^4^ cells/chamber in conditioned medium overnight, and then they were treated with DMSO (Control) and celastrol (1, 2, and 4 μM) for 8 h. The upper intermediary was substituted by conditioned intermediary, and the lower intermediary was substituted by intermediary supplemented with 20% FBS. Afterwards, the cells were subjected to fixation in 4% PFA for fifteen min and dyed with Gentian violet for ten min. The invaded cells were photographed with a microscopy device.

### Measurement of ROS production in cells

Cells were subjected to ROS-sensitive dye treatment, and the production of ROS within cells was detected via the flow cell technique. Briefly, three types of cells were placed into cultivation plates with 6 wells and cultivated overnight in standard cultivation intermediary. Afterwards, the cells were stimulated with celastrol at the indicated times and concentrations. The cells were dyed with 10 μM DCFH-DA under 37 °C for 0.5 h. The ROS status was identified via the flow cell technique (FACSCalibur, BD Biosciences, USA) and fluorescence images. In certain assays, the cells were subjected to 5 mM NAC pretreatment for 60 min. In the entire assays, 10,000 living cells were assayed.

### Cell apoptosis assay

The Annexin V-FITC Programmed Cell Death Identification Kit I and Propidium Iodide (PI) were bought from BD Pharmingen (America). The human NSCLC cells were inoculated into dishes (each with 6 wells) for the purpose of growing the cells to 80% confluency in the maximum medium, and afterwards, the cells were subjected to different concentrations of celastrol (1, 2, and 4 μM) and DMSO (Control) treatment for 48 h to assess the roles of celastrol in programmed cell death. In certain assays, the cells were subjected to 5 mM NAC pretreatment for 60 min. The cells were harvested and cleaned with cold PBS for 2 times, and afterwards, they were subjected to resuspension in binding buffering solution as per the specification of the Programmed Cell Death Kit. The cells were synchronously cultivated with luciferin-labeled Annexin V and PI. Afterwards, the Annexin V-binding buffering solution was added into the mix and the fluorescence property was identified on a FACSCalibur (USA). The Flowjo program was employed to analyze the figures.

### Hoechst 33342 staining

NSCLC cells were seeded in a six-well culture plate. After treatment with celastrol for 12 h, 4% PFA was used for 10 min for cell fixing, and the cells were cleaned in PBS, followed by Hoechst 33342 dyeing for 20 min. The characteristic of cell nucleus was observed by employing a fluorescence microscope (Leica, Tokyo, Japan).

### Determination of caspase 3 activity

Cells (1 × 10^6^ to 5 × 10^6^) were spread in petri dishes. Celastrol (1, 2, and 4 μM) was added to induce apoptosis in NSCLC cells. The cells were resuspended and lysed in 50 μL pre-treated lysis buffering solution for ten min on ice and then centrifuged for 1 min. The supernatant fraction was immediately transferred into a fresh tube. The reaction system was subsequently configured as specified in the Caspase 3 binding assay kit and maintained under 37 °C for 120 min. The absorbance was identified under 405 nm via a SpectraMax iD3 (USA).

### Western blot analysis

Tissue or cellular protein lysate buffering solution was utilized to perform protein extraction. Three cancer cell lines were seeded in the six plates, treated with celastrol for 3 h, and then dissolved with a cold lytic buffer. The 10% or 12% SDS-PAGE was used for protein separation. Proteins were translocated onto a PVDF film, followed by incubation for 1.5 h with 5% skimmed milk. The film was cultivated under 4 °C with specific antisubstances. Afterwards, the membrane underwent 1 h incubation at room temperature with the corresponding secondary antibody. Image J computer software was used for determining the density of the immune response bands.

### Cytoplasmic and nuclear protein extraction

We employed the NE-PER Nuclear and Cytoplasmic Extraction Kit to isolate the nucleus and cytoplasmic proteins in H460 cells (TFS). Cells were subjected to treatment with diverse concentrations of celastrol for 12 h, and afterwards, they were stimulated with IL-6 for 0.5 h. According to the protocol, the cell lysate was obtained. Immunoblotting assay was performed to identify the related protein expression.

### Immunofluorescence staining

H460 cells were inoculated into a fluorescence cuvette, and they were treated with celastrol and/or IL-6. Then, the cells were subjected to 4% PFA fixation and permeabilization via 0.5% Triton X-100 (in PBS), and cleaned three times with PBS. Afterwards, they were subjected to 1% BSA blockade for 60 min. Cell slides were cultivated with the first antisubstance (P-STAT3) in appropriate proportions. The cells were cultivated with an anti-rabbit secondary antisubstance (Alexa Fluor® 488) on the following day and then with the DAPI stain protected from light. The cell slides were cleaned in PBS again and subsequently sealed by anti-fluorescence quenching sealing tablets to prevent fluorescence quenching until they were captured by confocal microscopy (Nikon C2).

### Animal model

The WMU Animal Policy and Welfare Board accepted the entire murine assays. Athymic BALB/c nude mice (16–18 g) were bought from the Vital River Experimental Animal Center (PRC). For the heterograft model, H460 cells (5 × 10^6^ cells) blended with the same volume of PBS and Matrigel in 100 μL were delivered into the hind flank of the animals. If tumor volumes were ~ 50 mm^3^, the animals were separated into 4 experiment groups and they were injected with 4 mg/kg napabucasin (a recognized P-STAT3 suppressor) or celastrol (2 and 4 mg/kg) intraperitoneally every other day. Tumor volume was calculated as V = (L × W × W)/2, where L and W denote the length and width, respectively. The mice were euthanized on day 15, 2 h after the final drug administration. The tumors, hearts, livers, kidneys, and lungs were collected for the histological and immunoblotting assays.

### Immunohistochemical staining

Tumor samples were subjected to fixation under RT with 4% PFA and embedded with paraffin. The thickness of the treated tissues slices was 5 μm. After that, the samples were cultivated overnight with the proper primary antisubstance under 4 °C. The signal was identified via the relevant second antisubstance. Subsequently, these sections were dyed with DAB and counterstained with hematoxylin. Imaging was performed via optical microscopy.

### Hematoxylin and eosin (H&E) staining

The hearts, lungs, kidneys, and livers of mice were treated with 4% PFA fixation and paraffin embedment. The treated tumor sample slices (5 µM) were deparaffinized and subjected to rehydration, and afterwards, they were treated with H&E dyeing. Imaging was performed via optical microscopy.

### Statistical analysis

Data were presented as average ± SEM of three independently performed assays. The diversity in statistics between diverse groups was analyzed via the Student's t-test in GraphPad Pro7.0 (USA). *P* < 0.05 was deemed statistically significant.

## Results

### Celastrol suppressed the viability, migration, and invasion of NSCLC cells

**Fig. 1:**
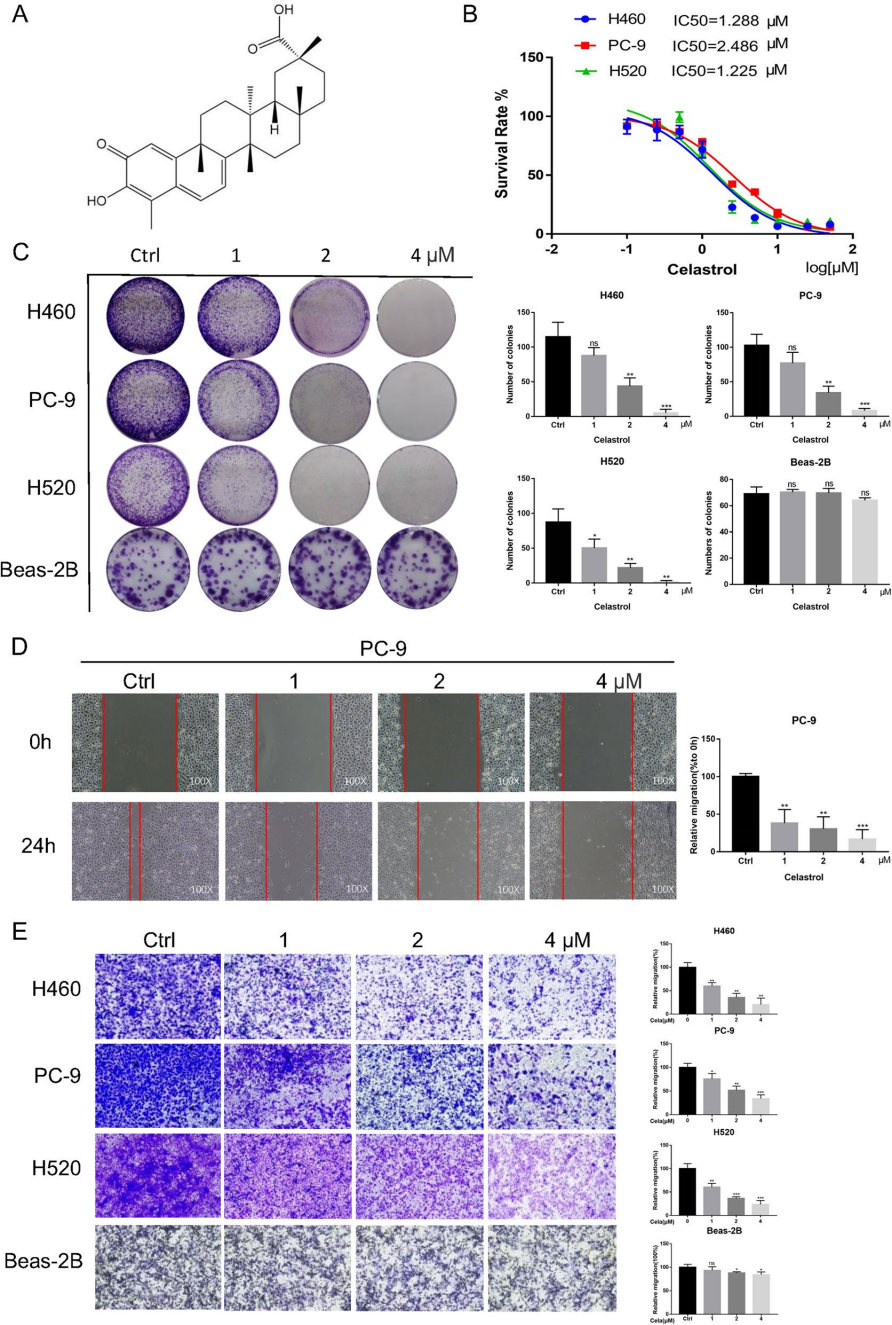
Celastrol inhibited the viability, migration, and invasion of NSCLC Cells. **A** Chemical structure of celastrol. **B** IC_50_ values of celastrol in NSCLC cells. Three NSCLC cell lines were placed in a 96-well plate with various concentrations of celastrol (0–100 μmol) After 48 h, treatment with celastrol, MTT assay was employed to determine the proliferation ability of cells. GraphPad Prism 7.0 software was used to acquire proliferation curves. **C** Representative images of colony formation assay. After incubation with the indicated dose of celastrol for 12 h, colonies of four cell lines were recorded after about 7 days. **D** PC-9 cells were treated with celastrol (0, 1, 2, and 4 μM) and allowed to migrate into the scratched area. **E** Celastrol induced loss of invasion potential in NSCLC cells. Triple assays were repeated. (*P < 0.05, **P < 0.01, ***P < 0.001)

The chemical structure of celastrol is shown in Fig. 1A. For the purpose of evaluating the suppressive role of celastrol, cellular activity was evaluated via the MTT analysis. Celastrol significantly inhibited the activity of H460, PC-9, and H520 cells, with IC_50_ values of 1.288 µM, 2.486 µM, and 1.225 µM, respectively (Fig. 1B). The cell counting trypan blue assay showed that the growth of H460 and PC-9 cells was significantly inhibited by celastrol in a concentration dependent manner (Additional file 1: Fig. S1A). Besides, the colony forming analysis revealed that celastrol could markedly inhibit the colony forming capability of NSCLC cells (Fig. 1C). Additionally, for the purpose of determining whether the treatment with celastrol was related to oncocyte migration and invasion, variations in migratory and invasive potency were identified via the wound healing assay and transwell assay; in contrast to controls, the migratory and invasive potency of these three lineage cells was dose-dependently inhibited by celastrol (Fig. 1D and E, and Additional file 1: Fig. S1B). At the same time, we conducted the same experiment in normal human cells. The results showed that celastrol had no obvious inhibitory effect on cell viability. To sum up, these results revealed that celastrol could validly suppress the viability, migration, and invasion of mankind NSCLC cells.

### Celastrol induced apoptosis of human NSCLC cells

**Fig. 2:**
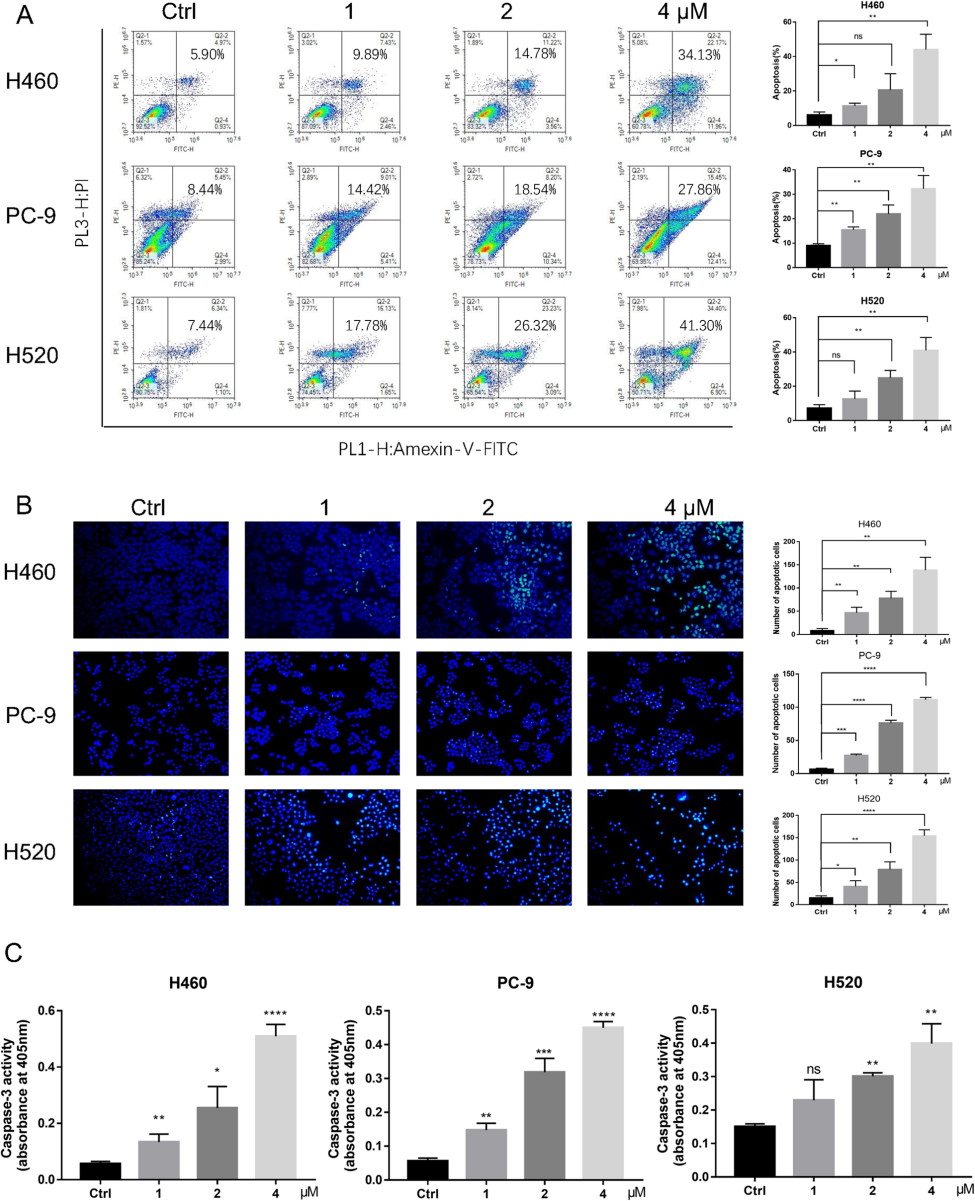
Celastrol induced apoptosis of human NSCLC Cells. **A** After treatment with celastrol at the indicated concentration for 24 h before staining with Annexin V and propidium iodide (PI), the distribution of apoptotic cells was determined using flow cytometry. **B** The apoptotic characteristics in NSCLC cell nucleus presented by Hoechst Staining assay. After treatment with celastrol for 12 h before staining with Hoechst staining, the nucleus was observed and photographed (100×). Representative results of triple experiments. **C** Caspase 3 activity was evaluated using the Caspase Assay Kit, and absorbance was recorded at 405 nm. (*P < 0.05, **P < 0.01, ***P < 0.001, ****P < 0.0001)

To assess whether celastrol can trigger programmed cell death, 3 lineage cells, H460, PC-9, and H520 were subjected to celastrol treatment at 3 diverse concentrations for 24 h, followed by staining with Annexin V FITC and PI, and the proportion of programmed cell death was identified via the flow cell technique. Celastrol markedly triggered programmed cell death in NSCLC cells (Fig. 2A). The outcomes of Hoechst 33342 dyeing showed that the celastrol-treated cells showed a potent blue fluorescent result and obvious apoptosis bodies. It was further confirmed that celastrol could induce apoptosis (Fig. 2B). Here, we also found that the activity of caspase 3 was significantly increased, as determined by the caspase activity assay, and the pro-apoptotic effect of celastrol was confirmed (Fig. 2C). Overall, celastrol markedly promoted apoptosis of NSCLC cells.

### Celastrol induced oxidative stress in human NSCLC cells

**Fig. 3:**
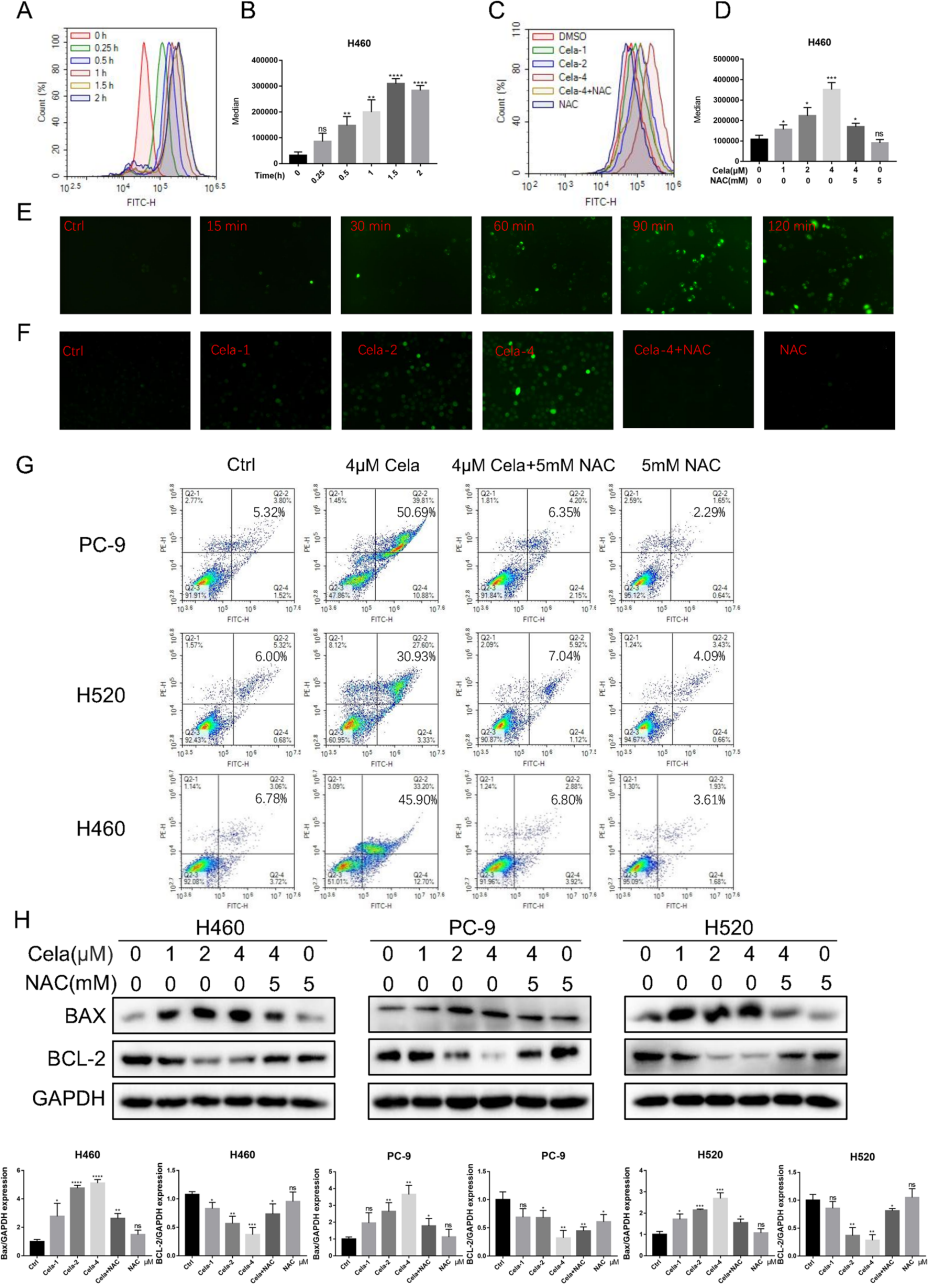
Celastrol induced oxidative stress in human NSCLC cells. **A**, **B** Intracellular ROS generation was measured by DCFH-DA. Cells were exposed to 4 µM celastrol for indicated times. Representative flow cytometric graph is shown in **A**. Quantification of ROS levels in H460 cells is shown in **B** (n = 3; *P < 0.05, **P < 0.01, ***P < 0.001 compared to 0 h control). **C**, **D**. Cells were treated with different concentrations of celastrol (0, 1, 2, and 4 μM) for 2 h. NAC was used at a dose of 5 µM, either alone or as a 1 h pretreatment. Representative flow cytometric graph is shown in **C**. Quantification of ROS levels in H460 cells is shown in **D** (n = 3; *P < 0.05, **P < 0.01, ***P < 0.001 compared to control). **E** Intracellular ROS as detected by DCFH-DA staining (green). H460 cells were exposed to 4 µM celastrol for indicated times and fluorescence images were captured. **F** H460 cells were treated as indicated in **C** and fluorescence images showing DCFH-DA (green) were captured. **G** Effect of NAC on celastrol-induced apoptosis. Cells were pretreated with 5 mM NAC for 1 h and then exposed to 4 µM celastrol for 24 h. The apoptotic cells were measured by Annexin V/PI staining. **H** Western blot analysis of BAX/BCL-2 in H460, PC-9, and H520 cells exposed to celastrol at indicated concentrations for 12 h. NAC pretreatment was carried out at 5 mM for 1 h. All experiments were repeated three times. (*P < 0.05, **P < 0.01, ***P < 0.001, ****P < 0.0001)

The production of ROS will cause programmed apoptosis. Promoting apoptosis by increasing ROS in tumor cells is one of the mechanisms of action of many chemotherapeutic agents [26, 27]. For the purpose of clarifying whether celastrol-triggered programmed cell death occurred due to ROS generation, we exposed H460, PC-9, and H520 cells to 4 µM celastrol and identified ROS via DCFH-DA dyeing. The numerical results herein revealed that celastrol remarkably elevated the ROS levels in NSCLC cell lines in a time-dependent manner (Fig. 3A, B and Additional file 2: Fig. S2A, B). Celastrol displayed dosage-reliant triggering of ROS in H460, PC-9, and H520 cells. As anticipated, the ROS signal was decreased in cells subjected to oxidation preventive NAC pretreatment (Fig. 3C, D and Additional file 2: Fig. S2C, D). ROS fluorescence images also showed that the intracellular green fluorescence increased rapidly with the time period of celastrol treatment (Fig. 3E and Additional file 2: Fig. S2E, F). Similarly, the intracellular green fluorescence accumulated with increasing drug concentration, and pretreatment with NAC could eliminate this phenomenon (Fig. 3F and Additional file 2: Fig. S2G, H). Afterwards, NAC was utilized to identify the participation of ROS in celastrol-triggered programmed cell death in NSCLC cells. Flow cytometry revealed that celastrol-triggered programmed cell death in H460 and PC-9, and H520 cells was reversed by 5 mM NAC pretreatment (Fig. 3G). These results were confirmed by the levels of apoptosis proteins, BAX and BCL-2 (Fig. 3H). Overall, the results indicated that an enhanced ROS level is the critical modulator of celastrol-triggered programmed cell death in NSCLC cells.

### Celastrol stimulated ER stress through ROS

**Fig. 4:**
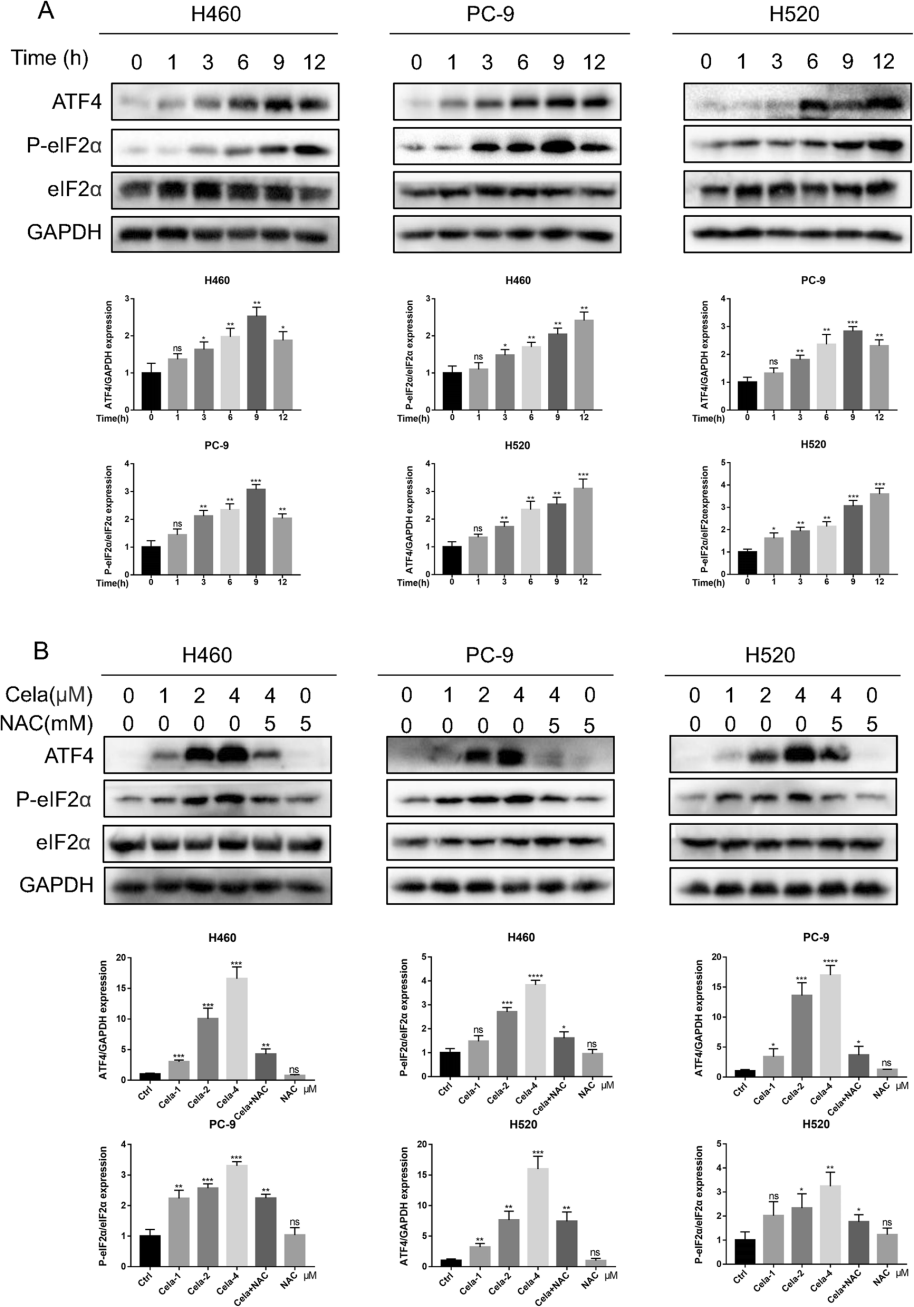
Celastrol activated ER stress through ROS. **A** Celastrol rapidly activates the ER stress pathway. H460, PC-9, and H520 cells were exposed to 4 µM celastrol for indicated times. Lysates were detected for p-eIF2α and ATF4. eIF2α and GAPDH was used as controls. **B** Effect of NAC on celastrol-induced ER stress. H460, PC-9, and H520 cells were pretreated with 5 mM NAC for 1 h and exposed to celastrol for 3 h. Western blot detected the lysates. Outcomes are representative of three independent experiments (*P < 0.05, **P < 0.01, ***P < 0.001, ****P < 0.0001)

Increased ROS levels have been found to trigger programmed cell death via various downstream pathways, such as ER stress and mitochondrion-related functional disorder [28]. Therefore, our team examined ER stress-related proteins, such as ATF4, P-eIF2α, and eIF2α, to determine if ROS levels elevated via celastrol triggered ER stress in NSCLC cells. These biomarkers of ER stress were rapidly increased in H460, PC-9, and H520 cells exposed to celastrol and this triggering occurred as early as 1 h after the treatment (Fig. 4A). Celastrol also dosage-dependently stimulated the production of ER stress-associated proteins, and after NAC pretreatment of NSCLC cells for 1 h, this induction was prevented (Fig. 4B). These research findings provide conclusive evidence that celastrol triggers ROS; thus, inducing ER stress and causing programmed cell death in NSCLC cells.

### Celastrol inhibited the IL-6/STAT3 signaling pathway in NSCLC cells

**Fig. 5:**
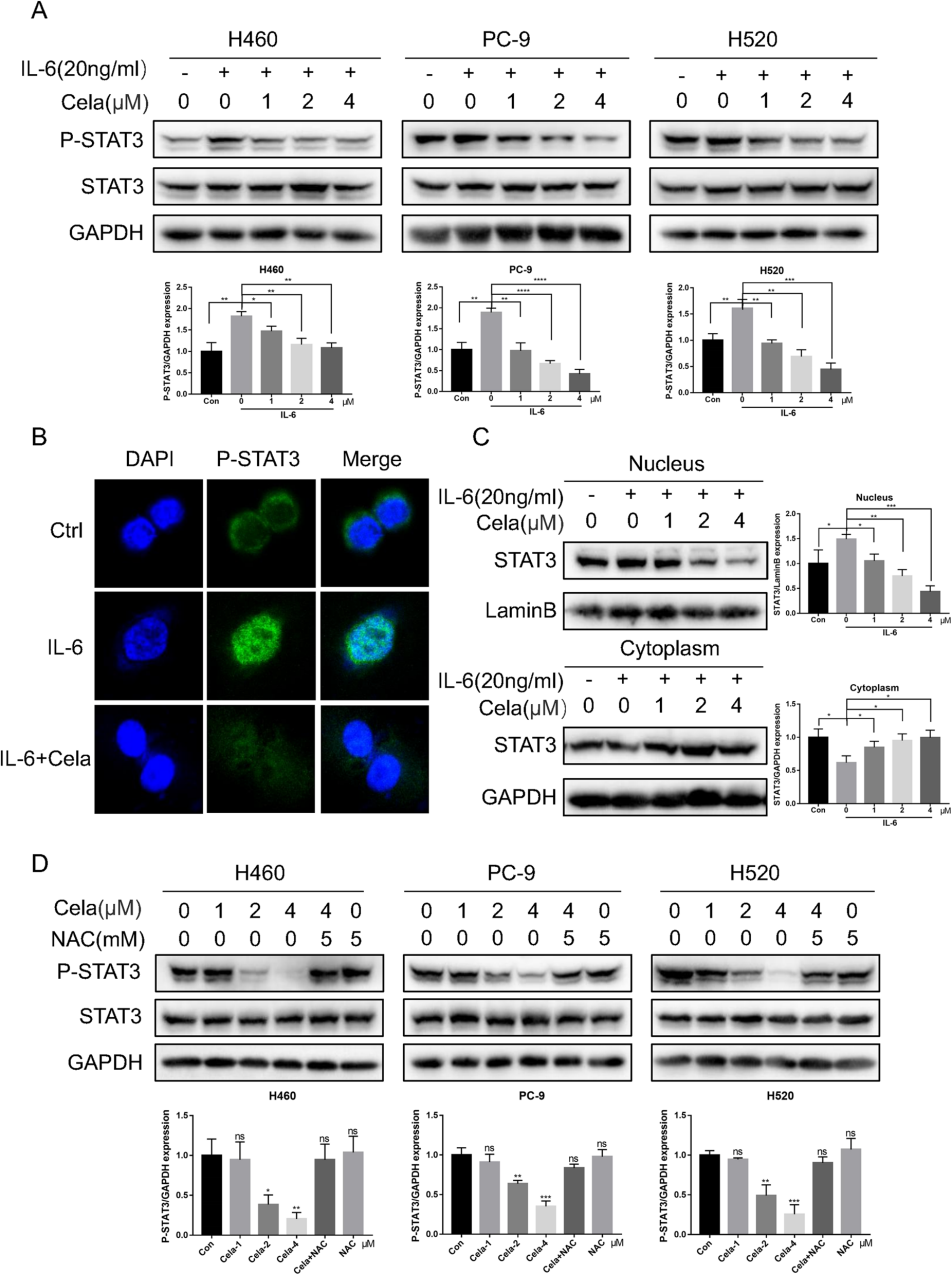
Celastrol inhibited the IL-6/STAT3 signaling pathway in NSCLC cells. **A** H460, PC-9, and H520 cells were exposed to IL-6 for 30 min after treatment with celastrol for 3 h, and the expression level of P-STAT3 was detected by western blotting. **B** Representative confocal microscopic images indicating the localization of p-STAT3 (green) and DAPI in H460 cells. **C** The expression levels of STAT3 in nuclear and cytosolic fractions were determined by using the western blot analysis. Images shown are representative of three separate experiments. **D** H460, PC-9, and H520 cells were treated with celastrol or in combination with NAC. The expression of p-STAT3 and STAT3 was detected by western blotting. Outcomes are representative of three independent experiments (*P < 0.05, **P < 0.01, ***P < 0.001, ****P < 0.0001)

Previous studies have reported that ROS regulate a variety of cell signal paths, and elevated ROS levels can inhibit the STAT3 pathway [21]. As a transcriptional factor, STAT3 is pivotal for oncocyte growth and development [29]. For this reason, our team reckoned whether celastrol could influence the IL-6/STAT3 signaling pathway in NSCLC cells. Cytokine IL-6 is the main inflammatory mediator and stimulator of STAT3, which can block programmed cell death and facilitate cancer survival. Therefore, our team used 20 ng/ml IL-6 and different concentrations of celastrol to treat H460, PC-9, and H520 cells. The result showed that the stimulation of STAT3 triggered by IL-6 was dosage-dependently reversed by celastrol (Fig. 5A). In addition, as per the immunofluorescence dyeing analysis, IL-6 therapy induced the cytoplasm-nucleus P-STAT3 transfer, which could be inhibited by celastrol (Fig. 5B). We used western blotting to detect the protein expression in the cytoplasm and nuclear extracts. These results further confirmed that the nuclear translocation of P-STAT3 was significantly inhibited by celastrol (Fig. 5C). We found that P-STAT3 in H460, PC-9, and H520 cells was markedly reduced with celastrol treatment; however, under the same treatment conditions, the expression of STAT3 did not change significantly. Interestingly, the inhibitory effect of celastrol-induced P-STAT3 was reversed after NAC pretreatment for 1 h in the three cell lines (Fig. 5D). Finally, we investigated the expression of ER stress related proteins after IL-6 and celastrol co-treatment, The experimental results are shown in the Additional file 2: Fig. S2I, The level of ER stress in IL-6 and celastrol co-treated group was higher than that in IL-6 treated group, but lower than that in celastrol treatment group, this suggests that IL-6 stimulated STAT3 phosphorylation can reduce the ER stress level caused by celastrol. This suggests that celastrol inhibits the STAT3 pathway by accumulating intracellular ROS. To sum up, these outcomes revealed that celastrol suppressed the IL-6/STAT3 pathway via ROS production in NSCLC cells.

### The therapeutic action of celastrol in vivo

**Fig. 6:**
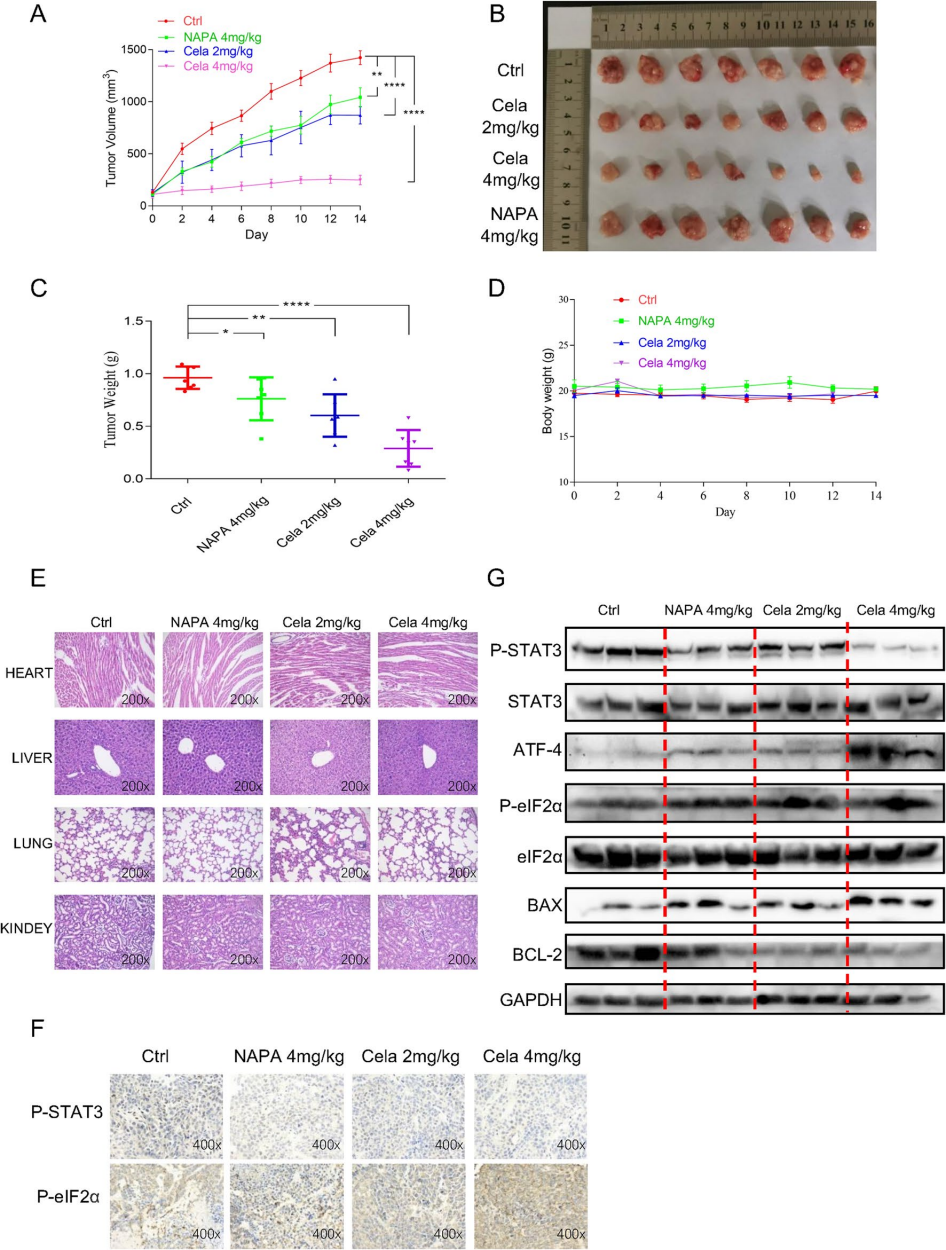
The therapeutic action of celastrol in vivo*. A* Tumor volumes. **B** Representative figures of the tumor tissue in the celastrol-administered and control groups (n = 7). **C** Measurement of tumor weights. **D** Body weight of mice. **E** Hematoxylin and eosin staining of hearts, livers, lungs, and kidneys from nude mice at 200× magnification. **F** The expression of P-STAT3 and P-EIF2α was detected by IHC in tumor tissues. **G** Representative blots indicating the expressions of P-STAT3, ATF4, P-eIF2α BAX, BCL-2, STAT3, eIF2α, and GAPDH in tumor tissues. The images shown are representative of three separate experiments (**P < 0.01, ***P < 0.001, ****P < 0.0001)

To further evaluate the anti-cancer efficiency of celastrol in vivo, we established and employed the cell-derived xenograft NSCLC model, and the H460 NSCLC cell line was selected for model establishment. The STAT3 suppressor napabucasin was utilized as the positive control. The mean volumes of tumors in the celastrol group (2 mg/kg or 4 mg/kg) were smaller compared to those in the control group and napabucasin (4 mg/kg) group (Fig. 6A), and the image of the tumors excised from nude mice after seven sessions of treatment with celastrol or napabucasin showed similar results (Fig. 6B). Consistent with the above data, the results shown in Fig. 6C indicated great reduction in the tumor weight after treatment with celastrol. Importantly, the body weight of mice did not decrease obviously (Fig. 6D), and the image of HE staining of vital organs (Heart, liver, kidney, and lung) did not present apparent toxicities (Fig. 6E). Thus, celastrol showed relative safety in vivo. As presented in Fig. 6F, the immunohistochemical results of NSCLC cells showed that, after injection of celastrol, the expression of P-STAT3 was decreased as the P-eif2α proteins increased (Fig. 6F). Western blotting showed that treatment with celastrol decreased the level of P-STAT3 and triggered the expression of apoptotic protein BAX; the BCL-2 level was inhibited. Meanwhile, ER stress induced by celastrol in the in vivo NSCLC model was also verified; the levels of ATF4 and P-eIF2α proteins were increased (Fig. 6G). Altogether, these results suggested that celastrol suppresses cancer development and cellular proliferative activity in vivo.

## Discussion

**Fig. 7:**
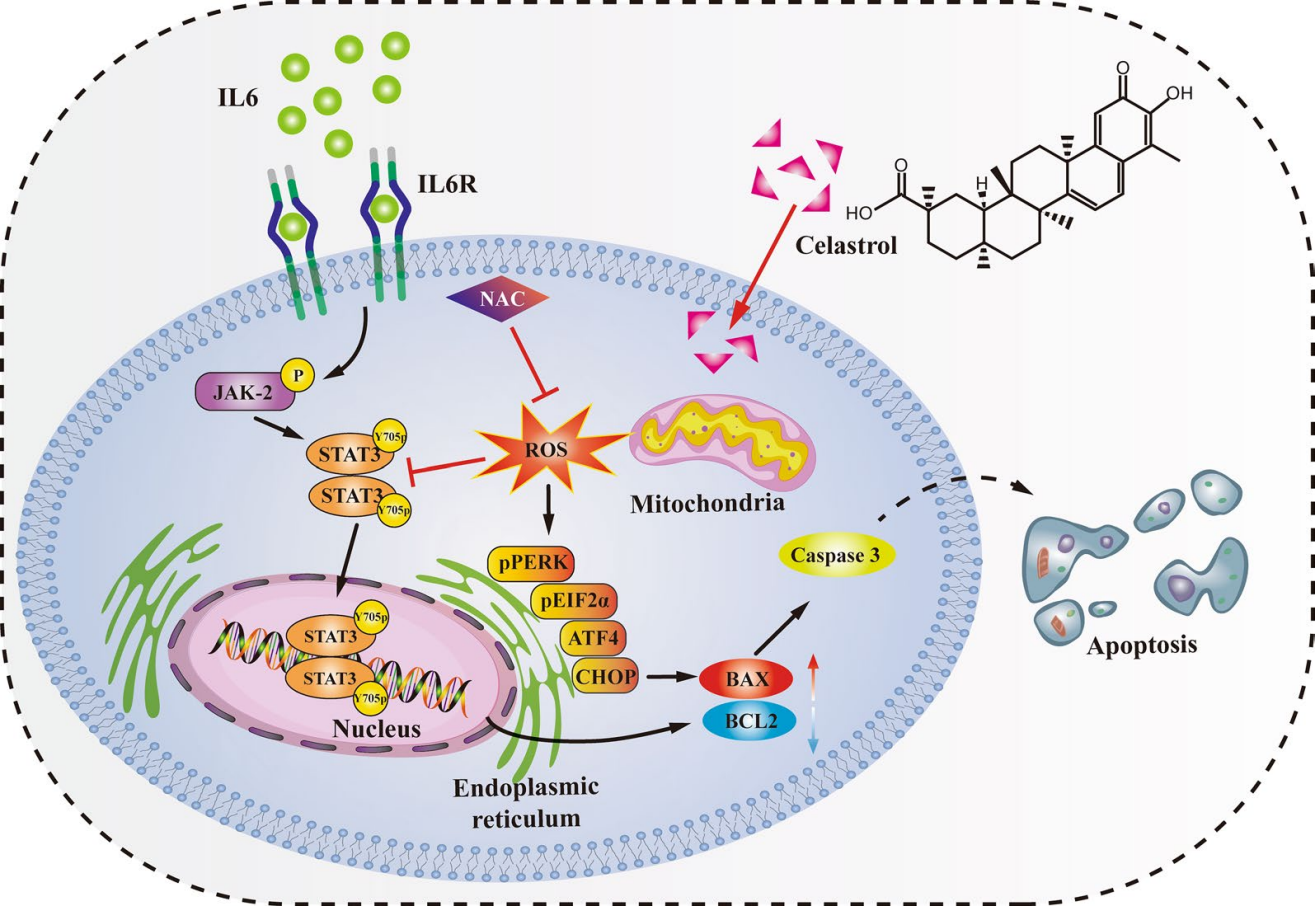
Mechanism of celastrol against NSCLC. Celastrol activated mitochondrial ROS to trigger endoplasmic reticulum stress and inhibited IL-6/STAT3 pathway to induce apoptosis in NSCLC

With an in-depth understanding of the molecular mechanisms of tumor genesis and development, it has become an important to discover active ingredients from natural products for the treatment of malignant tumors and clarify their mechanism of action [30–32]. In fact, several traditional chemotherapy drugs, including paclitaxel, epothilone, and vinca alkaloids, are derived from natural plants [32]. Celastrol is a triterpenoid compound extracted from Tripterygium wilfordii, a traditional Chinese medicinal herb. Celastrol has multiple biological pharmacological activities, which can not only inhibit inflammation and immune response, but can also enhance the sensitivity of the brain to leptin and inhibit hunger and obesity. In recent years, its anti-tumor activity has been reported [22, 33, 34]. Previous studies have found that celastrol has an inhibitory effect on a variety of NSCLC cell lines, but the specific mechanism of its anti-NSCLC effect is not clear [22, 35–37]. In this study, we found that celastrol could significantly inhibit the proliferation, clonogenesis, migration, and invasion of NSCLC cells, and cause excessive ER stress activation by significantly increasing the intracellular ROS level; thus, leading to the occurrence of apoptosis, while the ROS inhibitor NAC could completely reverse this effect. Further studies showed that celastrol could inhibit the STAT3 signaling pathway, and NAC could reverse the inhibitory effect of celastrol on the STAT3 signaling pathway. These results suggest that ROS may play a key role in the anti-NSCLC effect of celastrol (Fig. 7).

In this study, it was found that celastrol could induce apoptosis of NSCLC cells by flow cytometry and caspase-3 enzyme activity assay. Therefore, in order to determine whether celastrol-induced apoptosis is mediated by ROS, the DCFH-DA probe was used to label celastrol-treated cells. Intracellular ROS levels were detected by fluorescence microscopy and flow cytometry. The results showed that celastrol significantly increased the intracellular ROS levels in a time- and concentration- dependent manner. The apoptosis rate of NSCLC cells induced by celastrol was significantly reduced after the cells were pretreated with the antioxidant NAC. Western blot also showed that BAX protein expression was increased and BCL-2 protein expression was decreased in NSCLC cells treated with celastrol. NAC reversed this effect. These results suggest that celastrol induces apoptosis of human NSCLC cells through ROS-mediated oxidative stress injury.

The expression of ER stress-related proteins, P-eIF2α and ATF4, was significantly up-regulated in Western blot, indicating that celastrol induced ER stress in NSCLC cells. Previous studies have found that celastrol can induce a large amount of ROS production in NSCLC cells. In order to determine whether ER stress of NSCLC cells is induced by ROS, we added the antioxidant NAC to pretreat the cells. Western blot results showed that NAC significantly reduced the upregulation of ER stress-related proteins induced by celastrol. These results indicate that ROS may be the initiating factor of ER stress, and continuous and intense ER stress induces apoptosis of NSCLC cells.

Our study also showed that celastrol significantly inhibited STAT3 expression in a dose-dependent manner, even when stimulated by IL-6, the STAT3 activator. Previous studies have found that excessive accumulation of ROS in tumor cells can inhibit the STAT3 signaling pathway [20, 21]. Our study found that celastrol could significantly increase the ROS level in NSCLC cells and inhibit the STAT3 signaling pathway. In order to determine whether celastrol inhibits the STAT3 signaling pathway in NSCLC cells by ROS mediation, we tested STAT3 phosphorylation levels under different treatment conditions by Western blot, and the results showed that the antioxidant NAC could reverse the inhibitory effect of celastrol on the STAT3 signaling pathway. These results indicate that excessive accumulation of ROS induced by celastrol inhibits the STAT3 signaling pathway in NSCLC cells.

This study also evaluated the antitumor effect of celastrol in vivo, and found that celastrol reduced the volume and weight of NSCLC tumors. Celastrol inhibited the expression of P-STAT3 and promoted the expression of ATF4 and P-eIF2α. Celastrol up-regulated the expression level of BAX and down-regulated the expression level of BCL-2, which were consistent with the results of in vitro cell studies.

## Conclusion

In conclusion, our study indicates that celastrol has a significant inhibitory effect on NSCLC in vivo and in vitro, and ROS may be the key factor for celastrol to exert an anti-NSCLC effect. Celastrol can significantly increase the ROS levels in NSCLC cells, and then trigger ER stress and induce cell apoptosis. At the same time, high intracellular ROS level can also exert an anti-NSCLC effect by inhibiting the STAT3 signaling pathway. Celastrol has potential application prospects in drug therapy of NSCLC.

## Supplementary Information

**Additional file 1: Figure S1. A**. H460 and PC-9 cells were treated with different concentrations of celastrol (0, 1, 2, and 4 μM) for 24 h, after 10 min of trypan blue dyeing, viable and non-viable cells were counted in hemocytometer. Green represents viable cells, red represents non-viable cells. **B**. Representative Figures of H520 and Beas-2B cell migration assays. (*P < 0.05, **P < 0.01, ***P < 0.001).

**Additional file 2: Figure S2.** **A–B** and **E–F**. H460 and H520 cells were treated with 4 μM celastrol, and the levels of intracellular ROS were determined by flow cytometry and fluorescence microscope. **C–D** and **G–H**. H460 and H520 cells were treated with different concentrations of celastrol (0, 1, 2, and 4 μM) for 2 h. NAC was used at a dose of 5 μM, either alone or as a1h pretreatment, and the levels of intracellular ROS were determined by flow cytometry and fluorescence microscope. **I.** H460, and PC-9 cells were exposed to IL-6 for 30 min after treatment with 4 μM celastrol for 3 h, and the expression level of ATF4 and P- eIF2α was detected by western blotting, eIF2α and GAPDH was used as controls. (*P < 0.05, **P < 0.01, ***P < 0.001, ****P < 0.0001).

## Data Availability

The data set used and analyzed in this article would be available from the corresponding author on request.

## Abbreviations

BSA: Bovine serum albumin
CAS: Chinese Academy of Sciences
DAB: Diaminobenzidine
DCFH-DA: 2′,7′-Dichlorodihydrofluorescein diacetate
DMEM: Dulbecco’s Modified Eagle Medium
ER: Endoplasmic reticulum
IC_50_: Half-maximal inhibitory concentration
HE: Hematoxylin and eosin
NAC: N-acetyl-l-cysteine
OD: Optical density
PVDF: Polyvinylidene fluoride
PI: Propidium iodide
RPMI: Roswell Park Memorial Institute
RT: Room temperature
SIBS: Shanghai Institutes for Biological Sciences
STAT3: Signal and activator of transcription 3
TFS: Thermo Fisher Scientific
WMU: Wenzhou Medical University
