# Supplementary figures and images for: Celastrol elicits antitumor effects by inhibiting the STAT3 pathway through ROS accumulation in non-small cell lung cancer
Source: J Transl Med. 2022 Nov 12;20:525. doi: 10.1186/s12967-022-03741-9 (PMC9652895; doi:10.1186/s12967-022-03741-9)

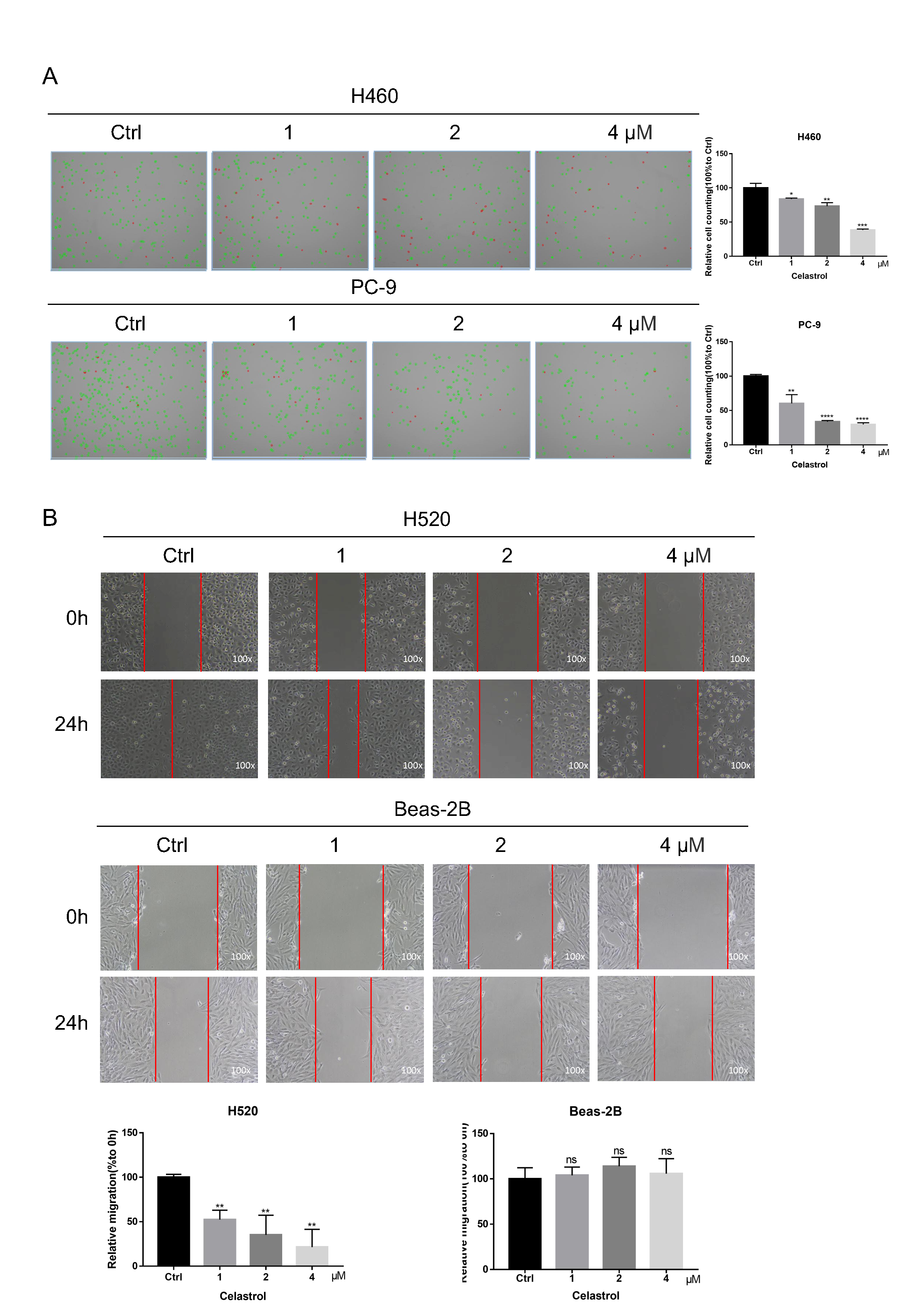

Supplement: Supplementary file 1 — Additional file 1: Figure S1. A. H460 and PC-9 cells were treated with different concentrations of celastrol (0, 1, 2, and 4 μM) for 24 h, after 10 min of trypan blue dyeing, viable and non-viable cells were counted in hemocytometer. Green represents viable cells, red represents non-viable cells. B. Representative Figures of H520 and Beas-2B cell migration assays. (*P < 0.05, **P < 0.01, ***P < 0.001). [file 12967_2022_3741_MOESM1_ESM.tiff]

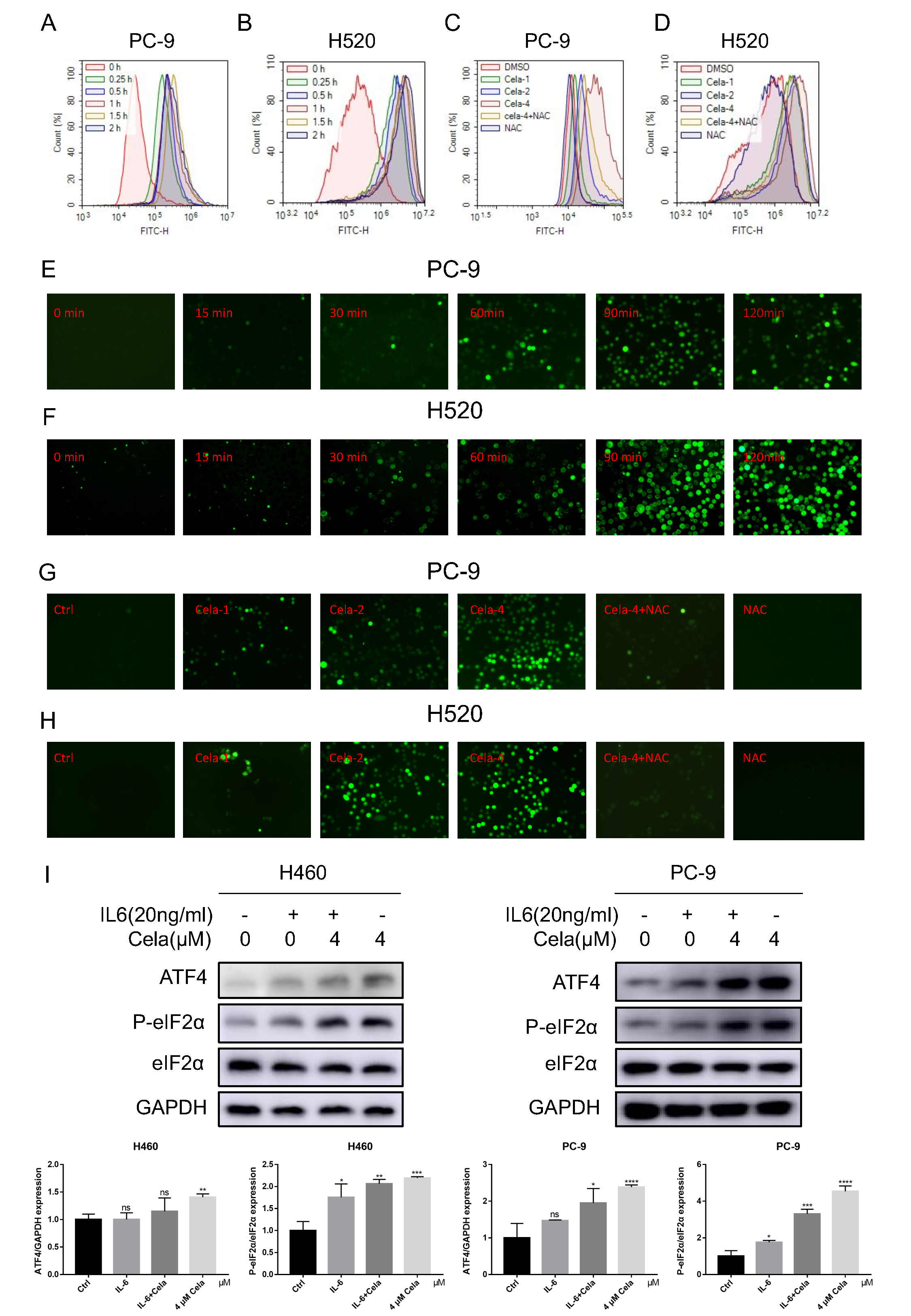

Supplement: Supplementary file 2 — Additional file 2: Figure S2. A–B and E–F. H460 and H520 cells were treated with 4 μM celastrol, and the levels of intracellular ROS were determined by flow cytometry and fluorescence microscope. C–D and G–H. H460 and H520 cells were treated with different concentrations of celastrol (0, 1, 2, and 4 μM) for 2 h. NAC was used at a dose of 5 μM, either alone or as a1h pretreatment, and the levels of intracellular ROS were determined by flow cytometry and fluorescence microscope. I. H460, and PC-9 cells were exposed to IL-6 for 30 min after treatment with 4 μM celastrol for 3 h, and the expression level of ATF4 and P- eIF2α was detected by western blotting, eIF2α and GAPDH was used as controls. (*P < 0.05, **P < 0.01, ***P < 0.001, ****P < 0.0001). [file 12967_2022_3741_MOESM2_ESM.tiff]
